# Supplementary material for: Intratumoral SIRPα-deficient macrophages activate tumor antigen-specific cytotoxic T cells under radiotherapy
Source: Nat Commun. 2021 May 28;12:3229. doi: 10.1038/s41467-021-23442-z (PMC8163884; doi:10.1038/s41467-021-23442-z)
Supplement: Supplementary file 1 — Supplementary Information [file 41467_2021_23442_MOESM1_ESM.pdf]

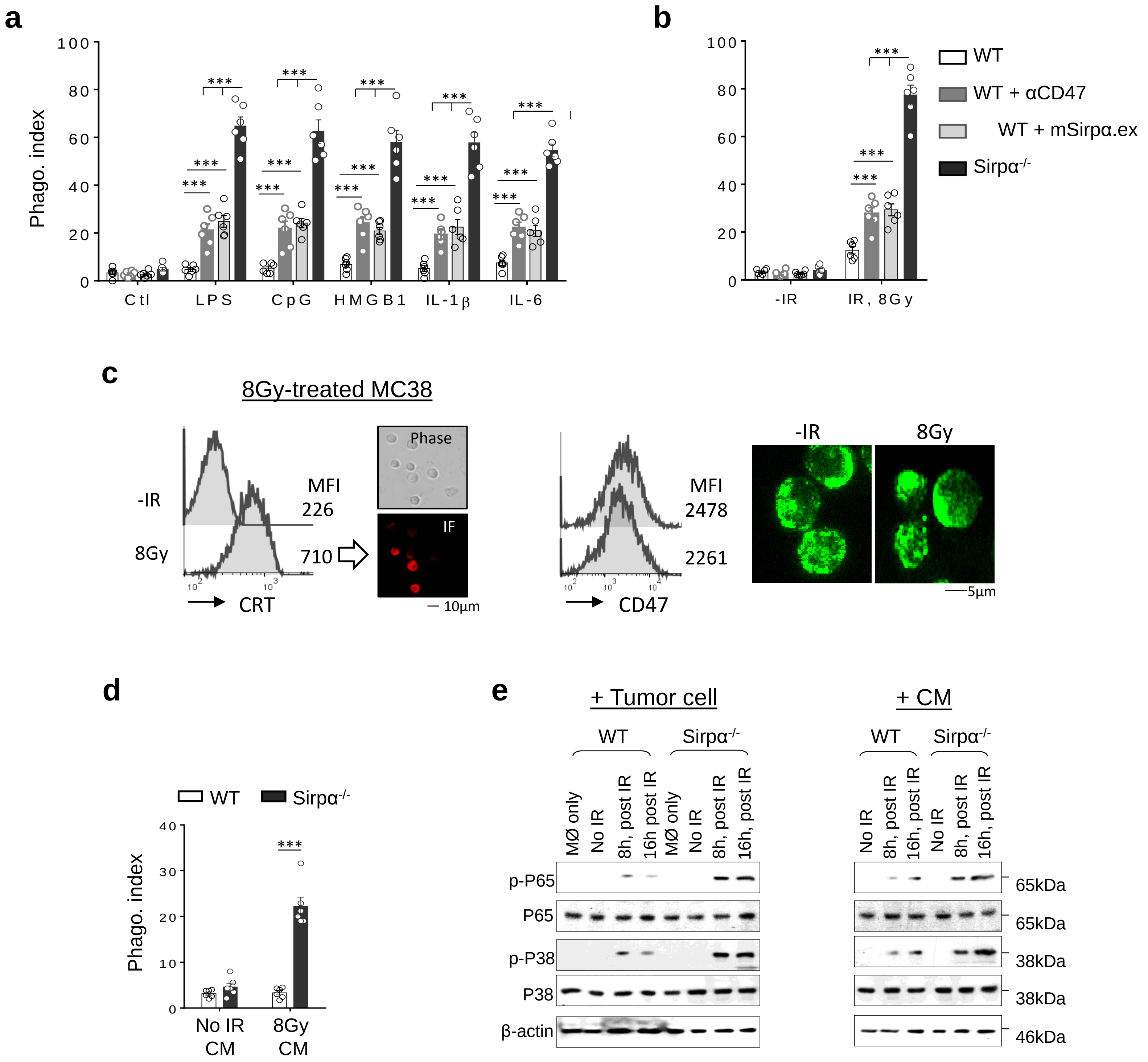

a
b
Phago. index
Ctl
LPS
CpG
HMGB1
IL-1β
IL-6
-IR
IR, 8Gy
WT
WT + αCD47
WT + mSirpα.ex
Sirpα-/-
c
8Gy-treated MC38
-IR
8Gy
MFI
226
710
CRT
Phase
IF
10μm
2478
2261
CD47
5μm
d
No IR CM
8Gy CM
e
+ Tumor cell
+ CM
MØ only
No IR
8h, post IR
16h, post IR
p-P65
P65
p-P38
P38
β-actin
65kDa
38kDa
46kDa

Supplementary Figure. 1. Phagocytosis of MC38 cells by activated Sirpα$^{-/-}$ macrophage. **a,** Phagocytosis of MC38 cells by LPS, CpG, HMGB1, IL-1β or IL-6 activated WT and Sirpα$^{-/-}$ BMDM in the presence or absence of CD47 antibody or SIRPα extracellular domain fusion protein (mSirpα.ex). Experiments were performed in duplicate and data from three independent experiments are presented as mean ± SEM. n = 6 per group. ***, $P < 0.001$; **b**, Phagocytosis of 8Gy-treated MC38 cells by WT and Sirpα$^{-/-}$ BMDM. Experiments were performed in duplicate and data from three independent experiments are presented as mean ± SEM. n = 6 per group. ***, $P < 0.001$ **c,** Immunostaining of calreticulin (CRT) and CD47 on non-treated and irradiated MC38 cells. Data are representative images from three independent experiments. **d,** Conditioned medium from irradiated MC38 cells induce Sirpα$^{-/-}$ macrophage to phagocytize MC38 cells. Experiments were performed in duplicate and data from three independent experiments are presented as mean ± SEM. n = 6 per group. ***, $P < 0.001$ **e,** Western blot of P65 and P38 in WT and Sirpα$^{-/-}$ BMDM after incubated with irradiated MC38 cells or their conditioned medium. Data are representative images from three independent experiments. *P* values were calculated by one-way ANOVA with Tukey's post hoc test. Source data are provided as a Source Data file.

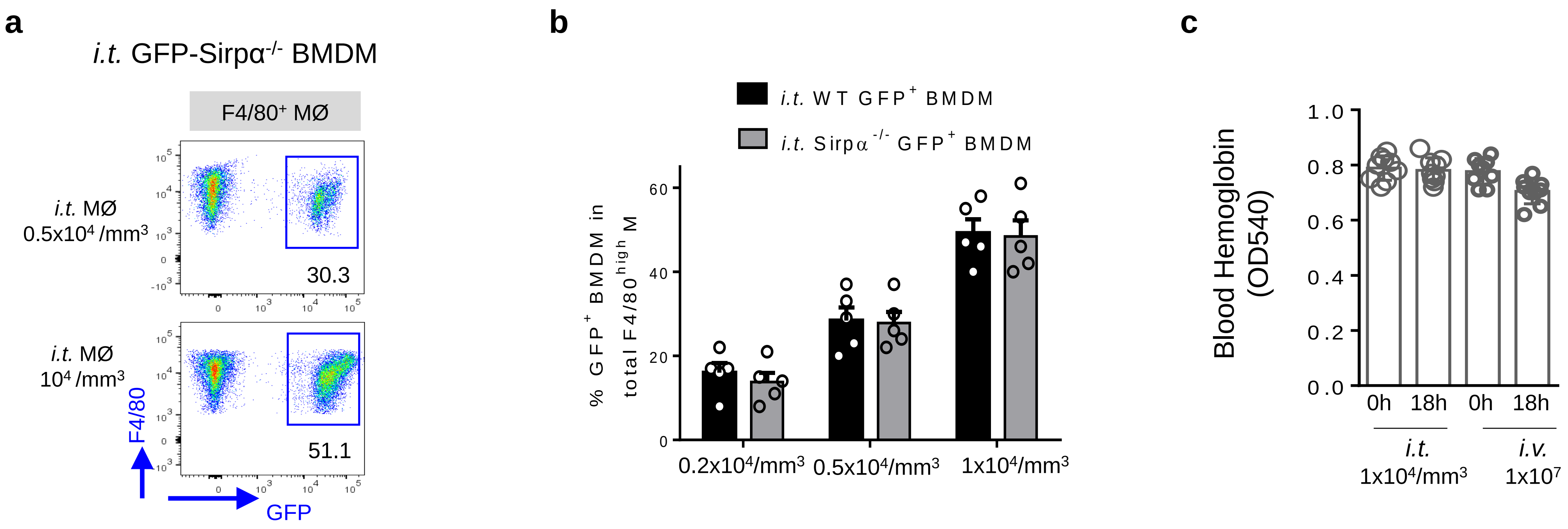

Supplementary Figure 2. Infusion of Sirpα$^{-/-}$ BMDMs into the engrafted tumor in WT mice. a-b) The frequency of infused GFP^{+} WT or Sirpα$^{-/-}$ BMDMs in MC38 tumors. GFP^{+} WT or Sirpα$^{-/-}$ BMDM were *i.t.* injected into MC38 tumor in WT recipient mice. The frequency of infused BMDM within F4/80^{high} TAM after 30mins of infusion were assessed by FACS (a) and summarized (b). n = 5 per group. c) Anemia analysis in mice 18 h post-Sirpα$^{-/-}$ BMDM infusion. Data from three independent experiments with 3 mice/group in each experiments are presented as mean ± SEM. n = 9 per group. Source data are provided as a Source Data file.

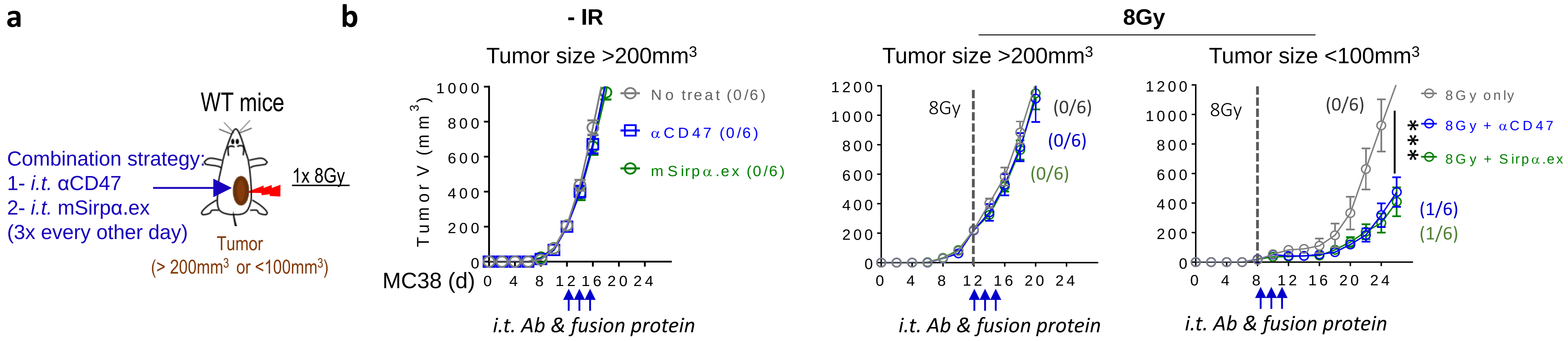

Supplementary Fig. 3. CD47 blockade alone or combination with IR failed to eliminate the well-established tumors. **a**, Depict of various combinations of treatments in tumor-bearing WT mice. Anti-CD47 antibody or mSIRPα.ex fusion protein was i.t. injected (50μg/mouse). **b**, Tumor growth in MC38-bearing WT mice after combining IR with CD47-blockade reagents. Data from two independent experiments (3 mice/group in each experiment) are presented as mean ± SEM. n = 6 mice in all groups. ***, $P < 0.001$; one-way ANOVA with Tukey's post hoc test. Source data are provided as a Source Data file.

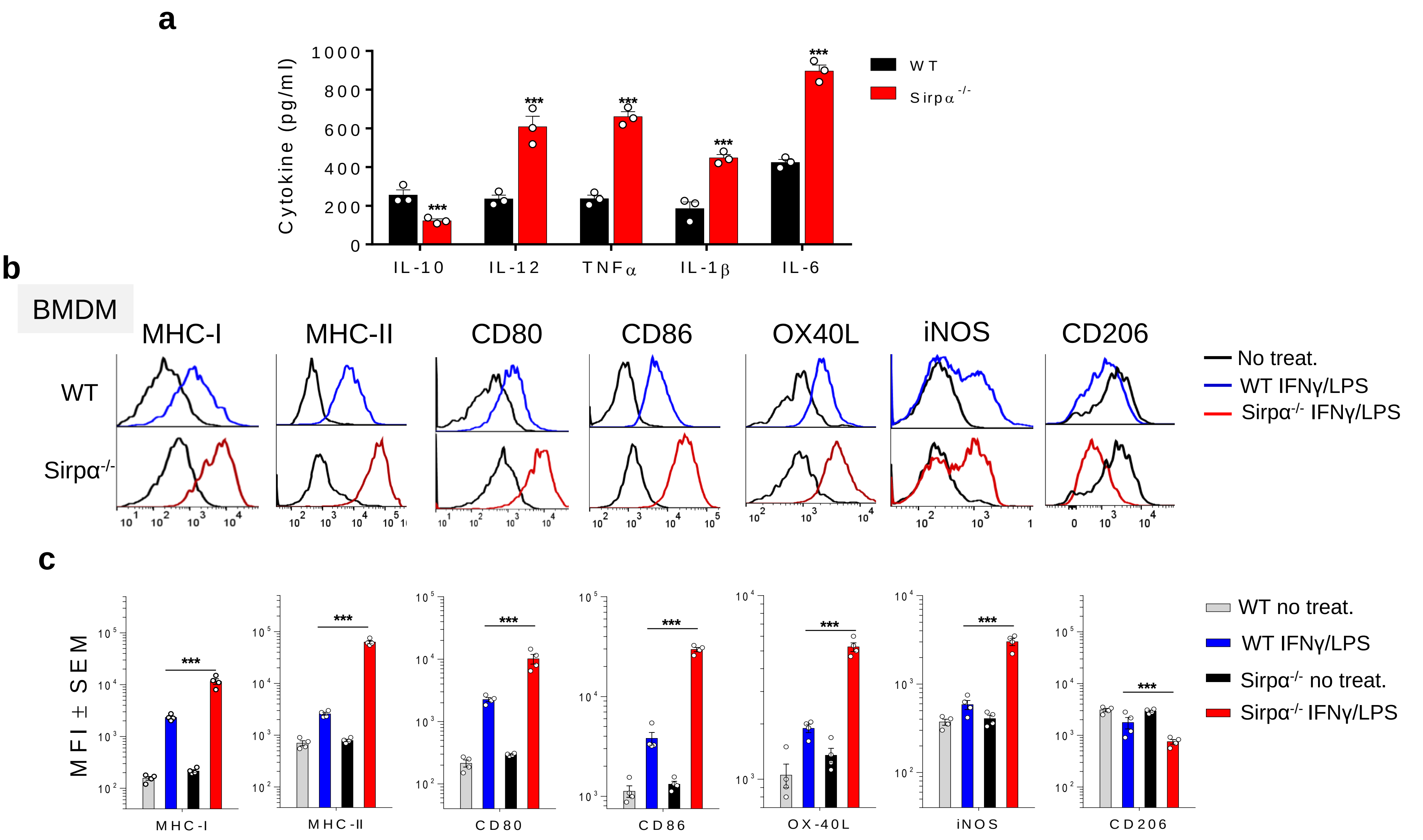

Supplementary Fig. 4. Enhanced expression of pro-inflammatory cytokines and antigen presentation related molecules in Sirpα$^{-/-}$ BMDM. **a**, Production of pro-and anti-inflammatory cytokines in WT and Sirpα$^{-/-}$ BMDM after IFNγ/LPS (20ng/ml each, 18h) treatment. ***, *P* < 0.001. n = 3 in all groups. **b-c**, Antigen presentation molecules and M1/M2 marker on WT and Sirpα$^{-/-}$ BMDM before and after IFNγ/LPS activation. Data from two independent experiments are presented as mean ± SEM. n = 4 in all groups. ***, *P* < 0.001. *P* values were calculated by one-way ANOVA with Tukey's post hoc test (a and c). Source data are provided as a Source Data file.

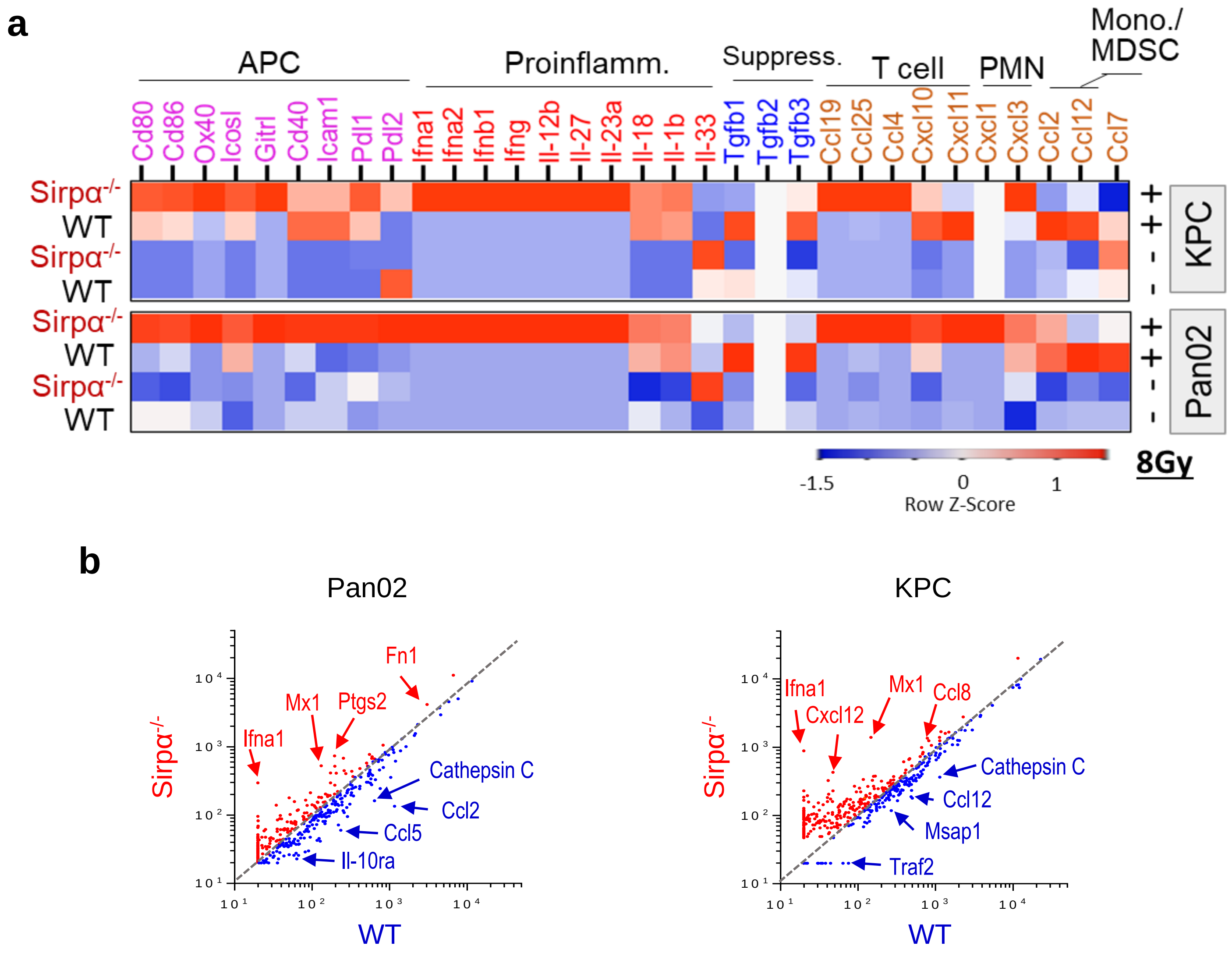

Supplementary Fig. 5. Nanostring analysis of mRNA expression profile in Pan02 and KPC tumors before and 12h post IR. Heatmap (**a**) and scatterplot (**b**) depicting differential expression of genes involved in antigen presentation, pro-inflammation, anti-inflammation and chemokines n = 4 mice in each group.

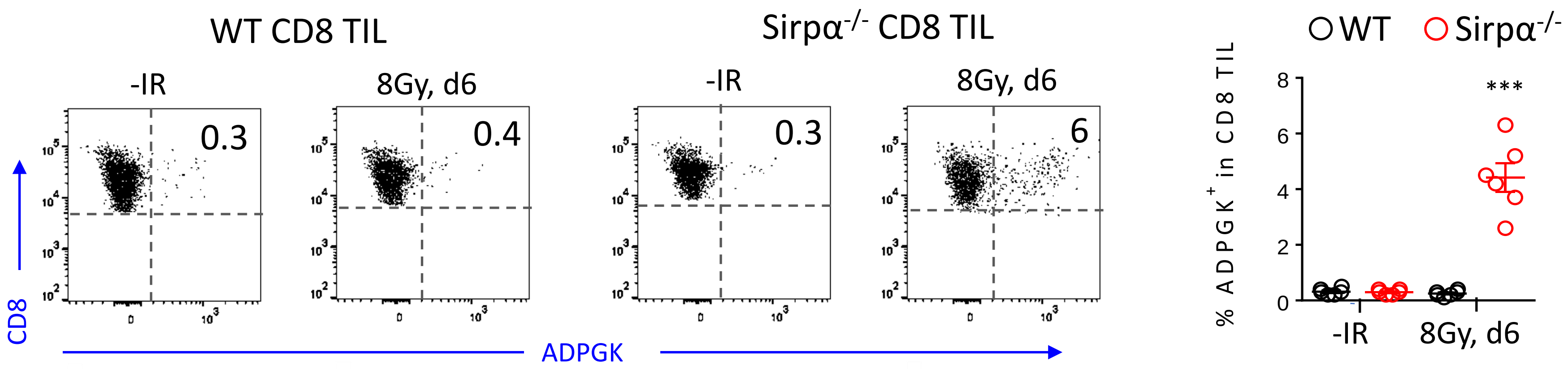

Supplementary Fig. 6. Percentage of ADPGK-tetramer^{+} cells in total CD8 TILs from WT and Sirpα$^{-/-}$ TME before and after IR. Left, representative flow cytometry image; Right, statistical results of percentage of ADPGK-tetramer+ cells in total TILs. Data from two independent experiments (3 mice/group in each experiment) are presented as mean ± SEM. n = 6 mice in all groups.***, $P < 0.001$; one-way ANOVA with Tukey's post hoc test. Source data are provided as a Source Data file.

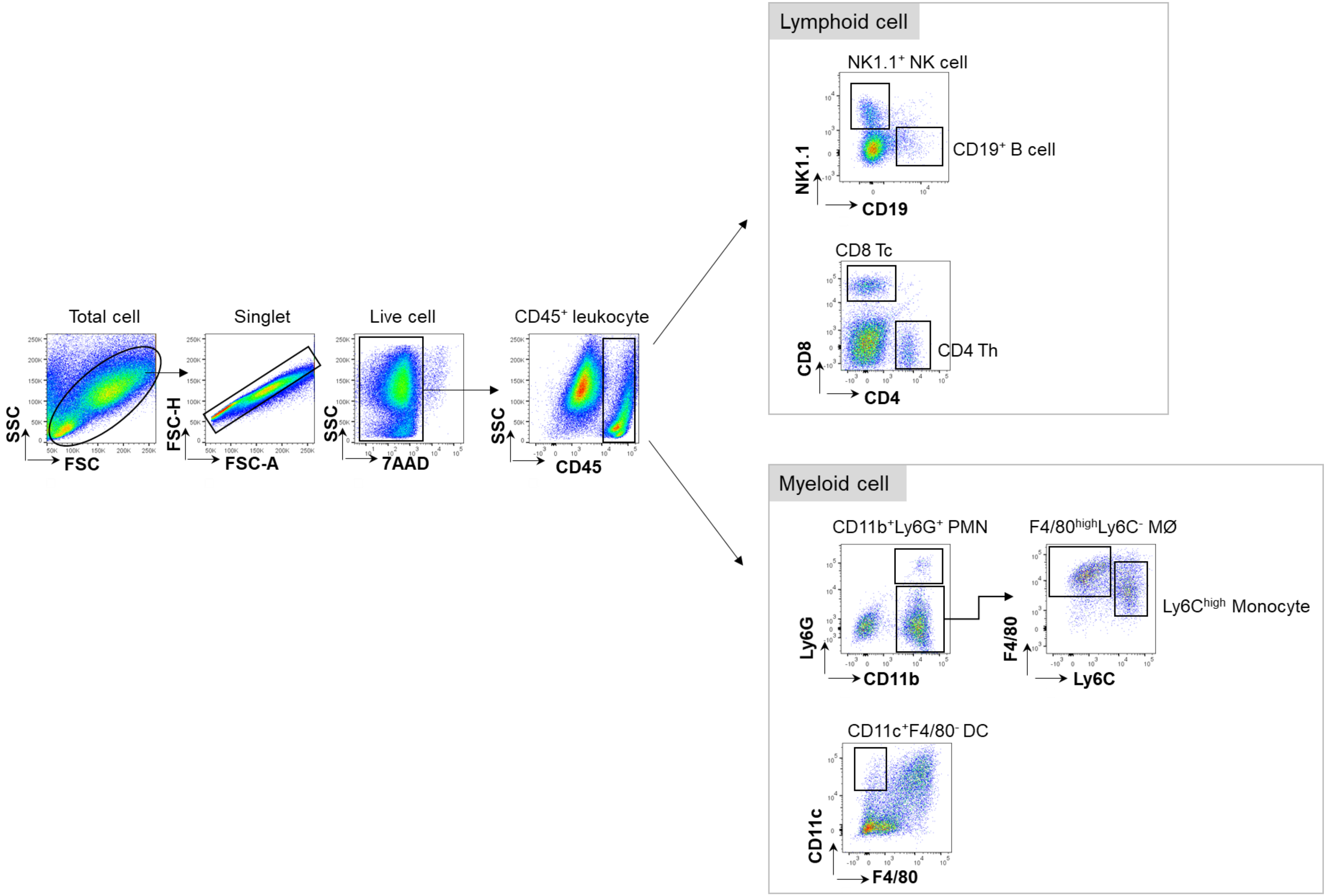

Supplementary Fig. 7. Gating strategy for flow cytometry analysis of leukocytes population in tumor microenvironment.

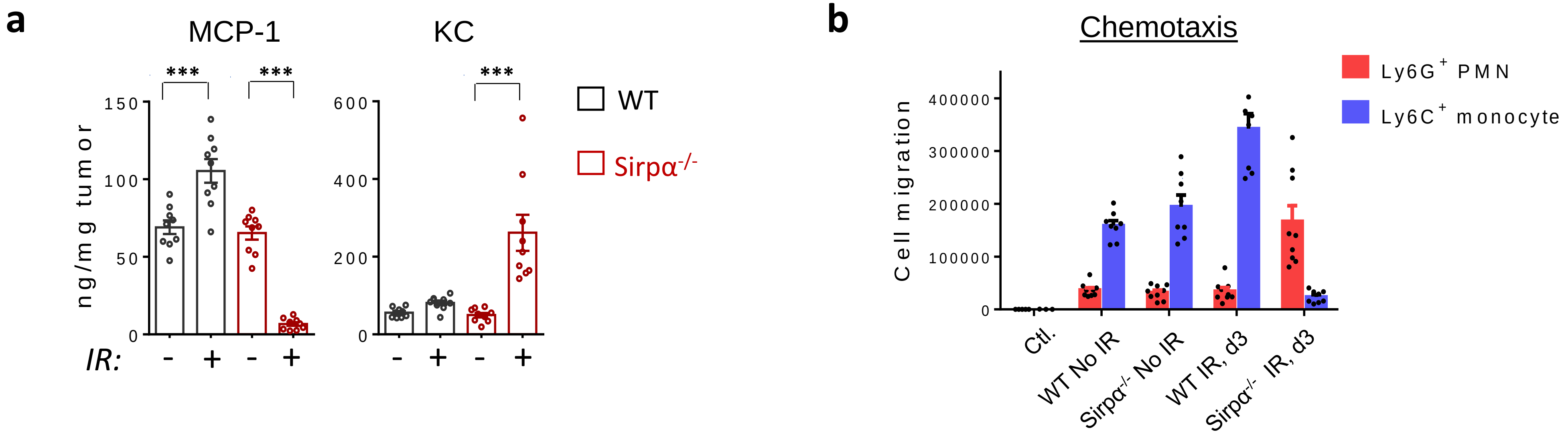

Supplementary Fig. 8. Chemokine analysis of conditioned medium of MC38 tumor isolated from WT and Sirpα$^{-/-}$ mice before and 3 days post-IR. **a**, Concentration of MCP-1 and KC in tumor conditioned medium from WT and Sirpα$^{-/-}$ MC38 tumor before and 3 days post-IR. **b,** Chemotaxis assay of Ly6C^{+} monocytes/monocytic MDSCs and Ly6G^{+} PMNs toward tumor conditioned medium. Data from three independent experiments are presented as mean ± SEM. n = 9 for Ly6G^{+} PMN, n = 8 for Ly6C^{+} monocyte. ***, $P < 0.001$; one-way ANOVA with Tukey's post hoc test. Source data are provided as a Source Data file.

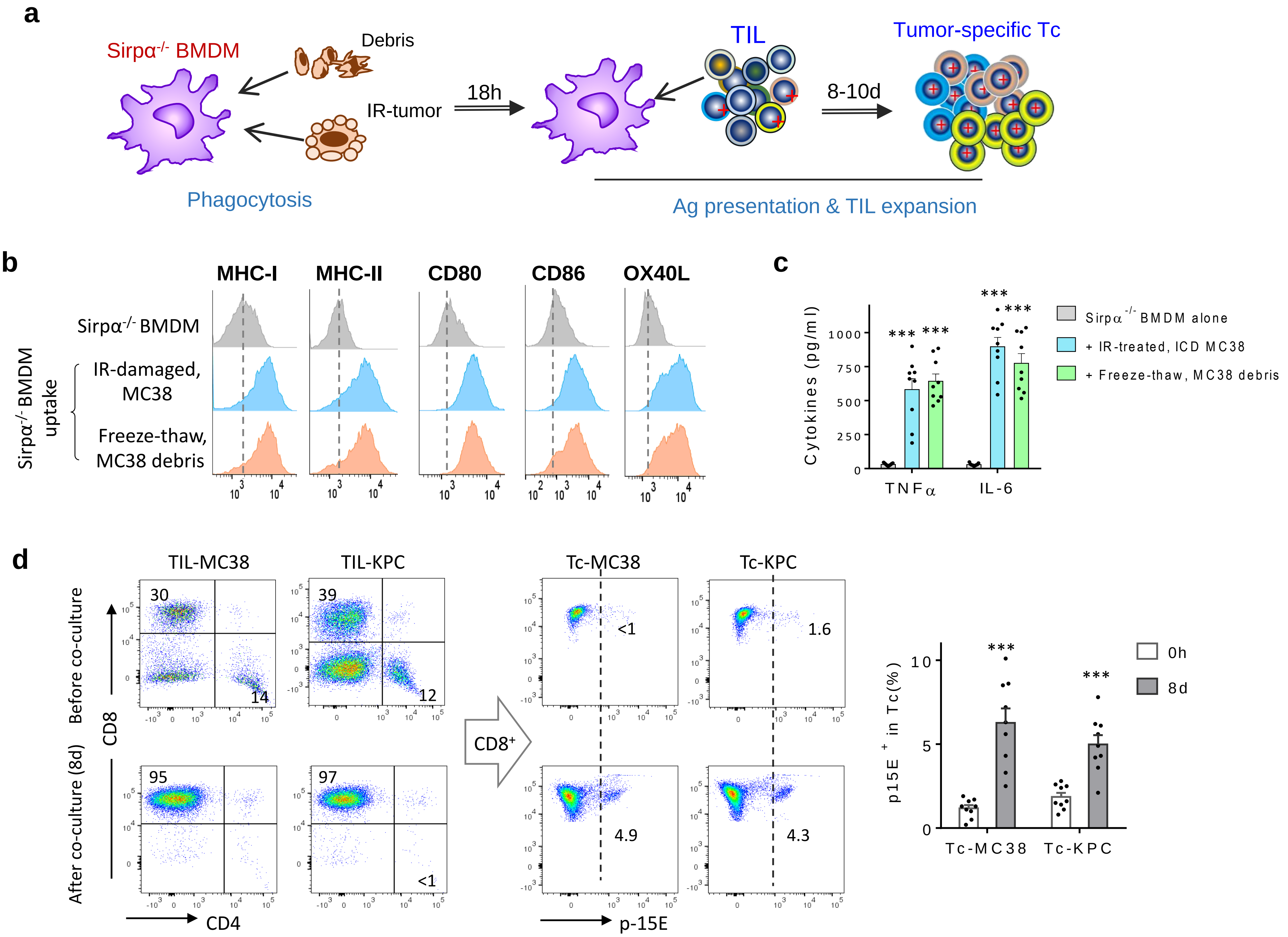

a
Sirpα-/- BMDM
Debris
IR-tumor
18h
TIL
8-10d
Tumor-specific Tc
Phagocytosis
Ag presentation & TIL expansion
b
MHC-I
MHC-II
CD80
CD86
OX40L
Sirpα-/- BMDM
IR-damaged, MC38
Freeze-thaw, MC38 debris
Sirpα-/- BMDM uptake
c
Cytokines (pg/ml)
TNFα
IL-6
Sirpα-/- BMDM alone
+ IR-treated, ICD MC38
+ Freeze-thaw, MC38 debris
d
TIL-MC38
TIL-KPC
Tc-MC38
Tc-KPC
Before co-culture
After co-culture (8d)
CD8
CD4
CD8+
p-15E
p15E+ in Tc(%)
0h
8d

Supplementary Figure. 9. Sirpα$^{-/-}$ BMDMs present tumor antigen to activate tumor-specific Tc cells. **a**, A schema of the experimental design. **b**, Upregulation of MHC-I, MHC-II, CD80, CD86 and OX40L on Sirpα$^{-/-}$ BMDM surface after tumor antigen uptake. Untreated Sirpα$^{-/-}$ BMDMs were used as negative control. Data are representative images from three independent experiments. **c**, Production of TNFα and IL-6 by Sirpα$^{-/-}$ BMDMs after tumor antigen uptake. Data from three independent experiments are presented as mean ± SEM. n = 9 in all groups. ***, $P < 0.001$ **d**, Frequency of p-15E Tetramer^{+} CD8 Tc cells before and after co-culture. TILs isolated from MC38 or KPC tumors were co-cultured with Sirpα$^{-/-}$ BMDMs loaded with corresponding tumor antigens. CD8 Tc before and after co-cultured were gated and frequency of p-15E Tetramer^{+} Tc cells were determined by FACS. Data from three independent experiments are presented as mean ± SEM. n = 9 in each group. ***, $P < 0.001$; one-way ANOVA with Tukey's post hoc test (**c** and **d**). Source data are provided as a Source Data file.

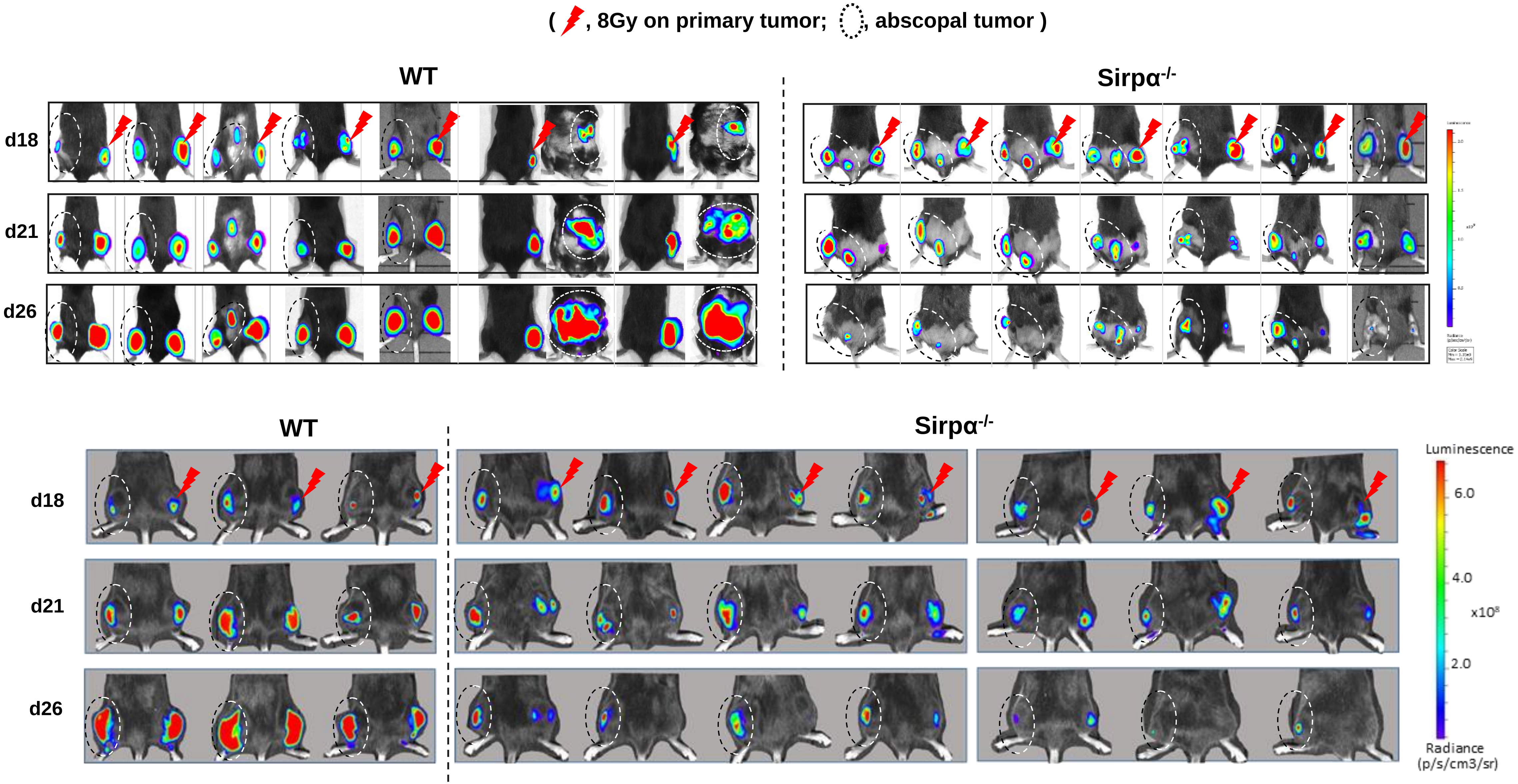

Supplementary Fig. 10. Supplemental images of Fig. 7

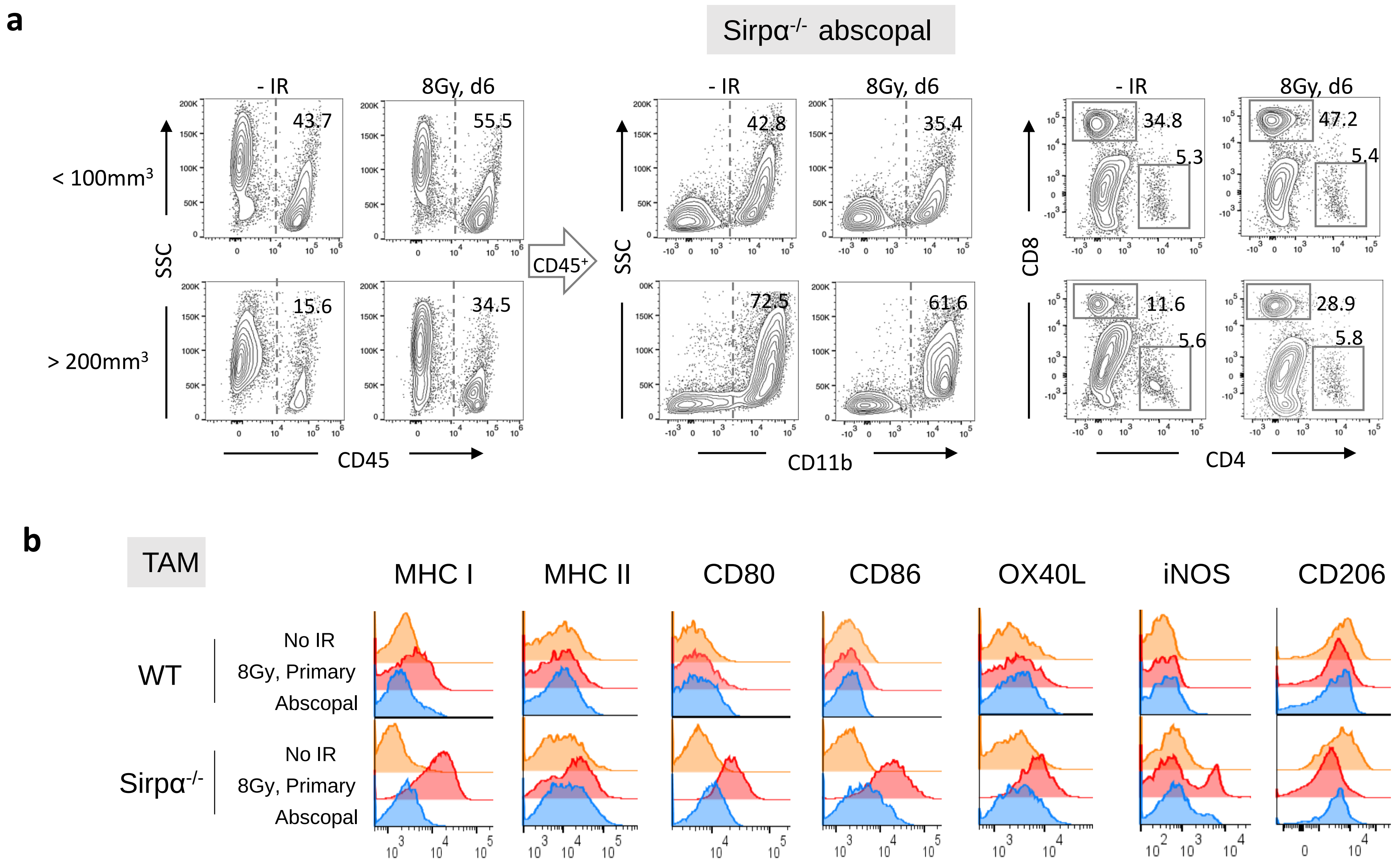

Supplementary Fig. 11. TME analysis of abscopal tumors. **a**, Leukocytes population in abscopal tumor before and after radiation of primary tumor. Primary tumors received 8Gy radiation when the abscopal tumors were <100mm^3 or >200mm^3. Leukocytes population including CD11b$^+$ myeloid cells, CD4$^+$Th and CD8$^+$ Tc within abscopal was analyzed by FACS. **b**, Expression of MHC I, MHC II, CD80, CD86, OX40L, iNOS and CD206 in abscopal TAM after radiation of primary tumor. Data are representative images from two independent experiments. n = 6 mice in each group.

Supplementary Table 1: Table of antibodies used in this study.

Name	Clone	Manufacture	Catalog Number	Dilution
FITC anti-mouse/human CD11b	M1/70	BioLegend	101206	1:100
FITC anti-mouse CD4	rm4-5	BioLegend	100510	1:100
FITC anti-mouse F4/80	BM8	BioLegend	123108	1:100
FITC anti-mouse Ly-6G	1A8	BioLegend	127606	1:100
FITC anti-mouse Ly-6C	HK1.4	BioLegend	128006	1:100
FITC anti-mouse I-A/I-E	M5/114.15.2	BioLegend	107606	1:100
FITC anti-mouse/human CD44	IM7	BioLegend	103006	1:100
FITC anti-human/mouse Granzyme B	QA16A02	BioLegend	372206	1:100
FITC anti-mouse CD86	GL-1	BioLegend	105006	1:100
PE anti-mouse H-2	M1/42	BioLegend	125506	1:100
PE anti-mouse CD8a	53-6.7	BioLegend	100708	1:100
PE anti-mouse Ly-6C	HK1.4	BioLegend	128008	1:100
PE anti-mouse CD252 (OX40 Ligand)	RM134L	BioLegend	108816	1:100
PE anti-mouse CD206	C068C2	BioLegend	141706	1:100
PE anti-mouse/human CD11b	M1/70	BioLegend	101208	1:100
PE anti-mouse CD4	GK1.5	BioLegend	100408	1:100
PE anti-mouse Ly-6G	1A8	BioLegend	127608	1:100
PE anti-mouse F4/80	BM8	BioLegend	123110	1:100
PE anti-mouse CD62L	MEL-14	BioLegend	104408	1:100
PE anti-mouse CD80	16-10A1	BioLegend	104708	1:100
Alexa Fluor® 594 anti-iNOS	W16030C	BioLegend	696804	1:100
APC anti-mouse CD80	16-10A1	BioLegend	104714	1:100
APC anti-mouse CD4	RM4-5	BioLegend	100516	1:100
APC anti-mouse F4/80	BM8	BioLegend	123116	1:100
APC anti-mouse CD11c	N418	BioLegend	117310	1:100
APC anti-mouse LY-6C	HK1.4	BioLegend	128016	1:100
APC anti-mouse IFN-γ	XMG1.2	BioLegend	505810	1:100
Brilliant Violet 650 anti-mouse F4/80	BM8	BioLegend	123149	1:100
Brilliant Violet 785 anti-mouse CD62L	MEL-14	BioLegend	104440	1:100
PE/DazzleTM 594 anti-mouse Ly-6C	HK1.4	BioLegend	128044	1:100
Pacific Blue anti-mouse CD45 Antibody	30-F11	BioLegend	103126	1:100
Biotin anti-mouse IL12/23 (p40)	C17.8	BioLegend	505302	1:100

Biotin anti-mouse IL-10	JES5-2A5	BioLegend	504906	1:100
Biotin anti-mouse IFN-β Antibody	MIB-5E9.1	BioLegend	508105	1:100
Anti-Mo/Rt FOXP3, eBioscience	FJK-16s	Invitrogen	14-5773-82	1:100
PE/Cy7 anti-mouse CD8a	53-6.7	BioLegend	100722	1:100
PerCP anti-mouse CD11c	N418	BioLegend	117326	1:100
PerCP/Cy5.5 anti-mouse NK-1.1	PK136	BioLegend	108728	1:100
PE/Cy7 anti-mouse CD19	6D5	BioLegend	115520	1:100
PE/Cy7 anti-mouse Ly-6G	1A8	BioLegend	127618	1:100
Pacific Blue anti-mouse/human CD44	IM7	BioLegend	103020	1:100
Pacific Blue anti-mouse CD11b	M1/70	BioLegend	101224	1:100
Mouse FcR Blocker	2.4G2	BioXCell	BE0307	1:100
InVivoMAb anti-mouse CSF1R	AFS98	BioXCell	BP0213	1:100
InVivoPlus anti-mouse CD4	GK1.5	BioXCell	BP0003-1	1:100
InVivoPlus anti-mouse CD8	2.43	BioXCell	BE0061	1:100
InVivoPlus anti-mouse PD-L1 (B7-H1)	10F.9G2	BioXCell	BP0101	1:100
InVivoMAb anti-mouse CD47 (IAP)	MIAP301	BioXCell	BE0270	1:100
PE-H-2Kb MuLV p15E Tetramer	N.A	MBL	TB-M507-1	1:100
PE-H-2Db Adpgk Neoepitope Tetramer	N.A	MBL	TB-5113-1	1:100
biotin-conjugated anti-mouse CD8a	53-6.7	BioLegend	100704	1:100
Streptavidin-PE	N.A	BioLegend	405204	1:100
Alexa488-conjagated anti-mouse IgG secondary antibody	N.A	Invitrogen	A28175	1:1000
Phospho-p38 MAPK (Thr180/Tyr182) XP® Rabbit mAb	D3F9	Cell Signaling	4511S	1:1000
p38 MAPK XP® Rabbit mAb	D13E1	Cell Signaling	8690S	1:1000
Phospho-NF-κB p65 (Ser536) Rabbit mAb	93H1	Cell Signaling	3033S	1:1000
NF-κB p65 XP® Rabbit mAb	D14E12	Cell Signaling	8242S	1:1000
β-Actin Rabbit mAb	1.30E+06	Cell Signaling	4970S	1:1000
Goat anti-Rabbit IgG (H+L) Secondary Antibody, HRP	N.A	Invitrogen	31460	1:10000
Calreticulin XP® Rabbit mAb	D3E6	Cell Signaling	12238S	1:100
Goat anti-Rabbit IgG (H+L) Secondary Antibody, PE	N.A	Invitrogen	P-2771MP	1:1000
