# Supplementary material for: Intratumoral SIRPα-deficient macrophages activate tumor antigen-specific cytotoxic T cells under radiotherapy
Source: Nat Commun. 2021 May 28;12:3229. doi: 10.1038/s41467-021-23442-z (PMC8163884; doi:10.1038/s41467-021-23442-z)
Supplement: Supplementary file 3 — Descriptions of Additional Supplementary Files [file 41467_2021_23442_MOESM3_ESM.pdf]

Descriptions of Additional Supplementary Files

Supplementary Movie 1

Description: TIL isolated from irradiated MC38 tumors in Sirpα-/- mice kill MC38 cells. MC38 tumors in Sirpα-/-mice were irradiated with a single fraction of 8Gy. Three days post-irradiation, CD8+ TIL were isolated from irradiated tumors and incubated with MC38 cells at a ratio of 1:4 (MC38:CD8+ TIL). Real-time videos recording TIL killing MC38 cells were made using a Nikon camera (DS-Qi1MC) with NIS-Elements software BR 4.20.00 (Nikon Instruments) that captures images at ten-second intervals over a three-hour period.

Supplementary Movie 2

Description: In vitro generated MC38-specific Tc kill MC38 cells. MC38-specific Tc generated by incubating MC38 TIL with MC38 tumor antigen-loaded Sirpα-/-macrophages were cocultured with MC38 cells at a ratio of 1:3 (MC38:CD8+ Tc). Real-time videos recording Tc killing MC38 cells were made using a Nikon camera (DS-Qi1MC) with NIS-Elements software BR 4.20.00 (Nikon Instruments) that captures images at ten-second intervals over a three-hour period.

Supplementary Movie 3

Description: In vitro generated KPC-specific Tc kill KPC cells. KPC-specific Tc were generated by incubating KPC TIL with KPC tumor antigen-loaded Sirpα-/-macrophages and then were cocultured with KPC cells at a ratio of 1:3 (KPC:CD8+ Tc). Real-time videos recording Tc killing KPC cells were made using a Nikon camera (DS-Qi1MC) with NIS-Elements software BR 4.20.00 (Nikon Instruments) that captures images at ten-second intervals over a three-hour period
