# Supplementary material for: Intratumoral SIRPα-deficient macrophages activate tumor antigen-specific cytotoxic T cells under radiotherapy
Source: Nat Commun. 2021 May 28;12:3229. doi: 10.1038/s41467-021-23442-z (PMC8163884; doi:10.1038/s41467-021-23442-z)
Supplement: Supplementary file 7 — Reporting Summary [file 41467_2021_23442_MOESM7_ESM.pdf]

nature research

Corresponding author(s): Yuan Liu

Last updated by author(s): Apr 19, 2021

Reporting Summary

Nature Research wishes to improve the reproducibility of the work that we publish. This form provides structure for consistency and transparency in reporting. For further information on Nature Research policies, see our Editorial Policies and the Editorial Policy Checklist.

Statistics

For all statistical analyses, confirm that the following items are present in the figure legend, table legend, main text, or Methods section.

n/a	Confirmed	
☐	☒	The exact sample size (n) for each experimental group/condition, given as a discrete number and unit of measurement
☐	☒	A statement on whether measurements were taken from distinct samples or whether the same sample was measured repeatedly
☐	☒	The statistical test(s) used AND whether they are one- or two-sided<br>*Only common tests should be described solely by name; describe more complex techniques in the Methods section.*
☐	☒	A description of all covariates tested
☐	☒	A description of any assumptions or corrections, such as tests of normality and adjustment for multiple comparisons
☐	☒	A full description of the statistical parameters including central tendency (e.g. means) or other basic estimates (e.g. regression coefficient) AND variation (e.g. standard deviation) or associated estimates of uncertainty (e.g. confidence intervals)
☐	☒	For null hypothesis testing, the test statistic (e.g. F, t, r) with confidence intervals, effect sizes, degrees of freedom and P value noted<br>*Give P values as exact values whenever suitable.*
☒	☐	For Bayesian analysis, information on the choice of priors and Markov chain Monte Carlo settings
☒	☐	For hierarchical and complex designs, identification of the appropriate level for tests and full reporting of outcomes
☒	☐	Estimates of effect sizes (e.g. Cohen's d, Pearson's r), indicating how they were calculated

Our web collection on statistics for biologists contains articles on many of the points above.

Software and code

Policy information about availability of computer code

Data collection	1. In vivo imaging data were collected using an IVIS Spectrum in vivo imaging system (Perkin Elmer).<br>2. Fluorescent microscopy was performed with All-in-one microscope (BZ-X700, Keyence).<br>3. FACS data were collected from BD LRSFortessa (BD Bioscience) using BD FACSDiva 8.0.2.<br>4. NanoString data collected by the Nanostring nCounter® SPRINT Profiler (NanoString Technologies).<br>5. Real-time videos recording Tc killing tumor cells were made using a Nikon camera DS-Qi1MC (Nikon Instruments).<br>6. Chemotaxis of CFSE labeled monocytes/MDSC and Ly6G+ PMN was quantified by a SpectraMax iD5 Multi-Mode Microplate Reader (Molecular Devices)
Data analysis	1. In vivo imaging datawere analyzed using Living Image software associated with IVIS Spectrum in vivo imaging system (Perkin Elmer).<br>2. BZ-X Analyzer 1.4.0.1 (Keyence) was used to analyze fluorescent images.<br>3. FACS data were analyzed using FlowJo 10.6 (Tree Star).<br>4. NanoString data were analyzed using nSolver 2.6 software (NanoString Technologies).<br>5. Real-time videos recording Tc killing tumor cells were analyzed using NIS-Elements software BR 4.20.00 (Nikon Instruments).<br>6. Chemotaxis of CFSE labeled monocytes/MDSC and Ly6G+ PMN was analyzed using SoftMax Pro 7 Software (Molecular Devices).<br>7. All graphs and statistical analyses were generated and performed using Prism 7.0 (GraphPad Software).

For manuscripts utilizing custom algorithms or software that are central to the research but not yet described in published literature, software must be made available to editors and reviewers. We strongly encourage code deposition in a community repository (e.g. GitHub). See the Nature Research guidelines for submitting code & software for further information.

Data

Policy information about availability of data

All manuscripts must include a data availability statement. This statement should provide the following information, where applicable:

- Accession codes, unique identifiers, or web links for publicly available datasets
- A list of figures that have associated raw data
- A description of any restrictions on data availability

All data included in this publication has been provided in the Source data file. Additional data support the findings of this study are available from the corresponding author upon reasonable request. NanoString gene expression profile has been deposited in the Gene Expression Omnibus (GEO) database (www.ncbi.nlm.nih.gov/geo, accession no. GSE149882).

Field-specific reporting

Please select the one below that is the best fit for your research. If you are not sure, read the appropriate sections before making your selection.

☒ Life sciences ☐ Behavioural & social sciences ☐ Ecological, evolutionary & environmental sciences

For a reference copy of the document with all sections, see nature.com/documents/nr-reporting-summary-flat.pdf

Life sciences study design

All studies must disclose on these points even when the disclosure is negative.

Sample size	No statistical method was used to predetermine sample size. The minimum number of animals used per treatment group was three, though six to eight animals were used per treatment group whenever mice were available.
Data exclusions	No data was excluded.
Replication	Experiments were successfully replicated, as specified in the figure legends.
Randomization	Mice were randomized into control or treatment groups when their tumor volume exceeded 150mm3 or 200mm3.
Blinding	Blinding was not possible due to the obvious phenotypic differences between wild-type and Sirpa-/- mice. However, each experiment was designed with appropriate controls and samples were collected under the same conditions for comparative analysis.

Reporting for specific materials, systems and methods

We require information from authors about some types of materials, experimental systems and methods used in many studies. Here, indicate whether each material, system or method listed is relevant to your study. If you are not sure if a list item applies to your research, read the appropriate section before selecting a response.

Materials & experimental systems

n/a	Involved in the study	
☐	☒	Antibodies
☐	☒	Eukaryotic cell lines
☒	☐	Palaeontology and archaeology
☐	☒	Animals and other organisms
☒	☐	Human research participants
☒	☐	Clinical data
☒	☐	Dual use research of concern

Methods

n/a	Involved in the study	
☒	☐	ChIP-seq
☐	☒	Flow cytometry
☒	☐	MRI-based neuroimaging

Antibodies

Antibodies used	All antibodies used in this study were obtained from commercial vendors. Their details (Supplier, conjugate, catalogue number) are described in Supplementary Table 1.
Validation	All antibodies were validated by their manufacturers for the application (flow cytometry, western blotting and immunohistochemistry). The validation data are available in the antibody data sheet on the vendor's websites. All antibodies were further validated in our lab by staining positive cells using protocols recommended manufactory.

Eukaryotic cell lines

Policy information about cell lines

Cell line source(s)	MC38 and Pan02 were from obtained from NCI. Luciferase-expressing KPC was a generous gift from Dr. Edmund Waller (Emory University)
Authentication	Mc38 and Pan02 were authenticated by the supplier. Luciferase-expressing KPC was authenticated by the expression of luciferase.
Mycoplasma contamination	All cell lines used in the study were negative for mycoplasma contamination.
Commonly misidentified lines (See ICLAC register)	None of the cell lines used in this study were commonly misidentified lines listed in ICLAC.

Animals and other organisms

Policy information about studies involving animals; ARRIVE guidelines recommended for reporting animal research

Laboratory animals	Age- and sex-matched male and female adult (8-16-week-old) Sirpα-deficient or Sirpα-deficient-GFP mice and their wild-type (WT) littermates were used in each independent experiment. The generation and characterization of Sirpα-/- mice have been described in our previous study. Sirpα-deficient-GFP mice were generated by crossing Sirpα-deficient mice with C57BL/6-Tg (UBC-GFP) 30Scha/J (The Jackson Laboratory). Mouse were housed in an institutional pathogen-free facility on a 12-h reverse light/dark cycle (7PM-7AM). The animal facility was maintained at a temperature of 23 ± 2°C with 55 ± 5% humidity.
Wild animals	No wild animals were used in this study
Field-collected samples	No field-collected samples were used in this study.
Ethics oversight	All experiments using animals and procedures of animal care and handling were carried out following protocols approved by the Institutional Animal Care and Use Committee (IACUC) of Georgia State University.

Note that full information on the approval of the study protocol must also be provided in the manuscript.

Flow Cytometry

Plots

Confirm that:

- [x] The axis labels state the marker and fluorochrome used (e.g. CD4-FITC).
- [x] The axis scales are clearly visible. Include numbers along axes only for bottom left plot of group (a 'group' is an analysis of identical markers).
- [x] All plots are contour plots with outliers or pseudocolor plots.
- [x] A numerical value for number of cells or percentage (with statistics) is provided.

Methodology

Sample preparation	To analyze tumor-associated leukocytes, excised tumors were minced with sterilized scissors and were dissociated using the GentleMACS Dissociator with Mouse Tumor Dissociation Kit (Miltenyi Biotech) according to the manufacturer's instructions with modifications. Briefly, after digestion with the provided enzyme mixture (45 min, 37°C) and harvesting dissociated cells, the remaining tissues were further digested with 10% (v/v) trypsin containing 5 mM EDTA in Hank's balanced salt solution without calcium and magnesium for 15 min at 37°C to improve recovery of macrophages and other myeloid leukocytes. The dissociated cells in suspension were then filtered through a 70µm nylon strainer, treated with red blood cell lysis buffer and were counted by a cell counter. For flow cytometric analyses, cells were blocked with anti-FcR (clone 2.4G2; Bio X Cell) before incubating with a mix of fluorophore-conjugated antibodies (4°C, 30min) to detect different populations of tumor-associated leukocytes (gated as CD45+) that included macrophages (gated as CD45+CD11b+F4/80highLy6C-), monocytes (CD45+CD11b +F4/80+ Ly6Chigh), granulocytes/PMN (CD45+CD11b+ Ly6Ghigh), dendritic cells (DC, CD45+CD11c+F4/80-), CD4 Th (CD45 +CD4+), CD8 Tc (CD45+CD8+), NK (CD45+NK1.1+) and B cells (CD45+CD19+). Dead cells were excluded by 7AAD staining (Gating strategy also see Extended Data Fig. 7). Antibodies were from BioLegend, eBioscience and BD Pharmingen. CD8 Tc that recognize the tumor-specific antigens p15E and ADPGK were detected by H-2Kb MuLV p15E Tetramer and H-2Db ADPGK Neoepitope Tetramer (both from MBL Life Science), respectively. Effector memory T cells within CD8 Tc population were gated as CD44+CD62L-. Treg and Th1 cells within CD4 Th were gated as Foxp3+ and IFNγ+, respectively, after intracellular staining (Intracellular Cytokine Staining kit from BD Pharmingen was used for detecting IFNγ). CD8 Tc and NK cells expressing granzyme B (GranzB) were detected by intracellular staining with a mouse anti-human/mouse granzyme B antibody (BioLegend). To detect PMN expressing ROS, total dissociated cells were treated with 1µM PMA (4 h, 37°C) in the presence of oxidation-sensitive dye DCFDA (5µM, Invitrogen) followed by washing and flow cytometry48,49. To detect macrophage expression of cytokines, total dissociated cells were treated with brefeldin A (BioLegend, 5 µg/ml) for 4 h at 37°C and cell surface staining for cell type gating, followed by fixation and permeabilization and intracellular staining using cytokine-specific fluorophore-conjugated antibodies or biotin-conjugated primary antibodies in combination with streptavidin-PE.

Instrument	BD LRSFortessa (BD Bioscience) withBD FACSDiva 8.0.2.
Software	Flow cytometry data were analyzed using FlowJo 10.6.
Cell population abundance	Expressed as a frequency of the selected population
Gating strategy	The FMOs were used for determining the division for negative and positive population. Detailed gating strategy was shown in Extend Data Figure 7.

☒ Tick this box to confirm that a figure exemplifying the gating strategy is provided in the Supplementary Information.
